# Biological Safety of 3D Printing Materials Based on Acrylic Resins Used in Dentistry: Narrative Review

**DOI:** 10.3390/ma19091905

**Published:** 2026-05-06

**Authors:** Małgorzata Ponto-Wolska, Zbigniew Raszewski

**Affiliations:** 1 Department of Dental Propaedeutics and Prophylaxis, Faculty of Medicine and Dentistry, Medical University of Warsaw, 02-006 Warsaw, Poland; 2 Prosthetic Department, WSM Białystok, ul. Sobieskiego 4, 15-027 Białystok, Poland

**Keywords:** 3D printing, methacrylic resin, cytotoxicity, allergic reaction, biological properties

## Abstract

**Highlights:**

- 3D resins can be used safely in dentistry to make different types of restorations following the manufacturer’s specifications.
- Polymerization, washing with alcohol and post-polymerization need to be performed in a light oven.
- The biological response to organisms is caused by non-polymerized methacrylic monomers, eluting catalysts and stabilizers present in the materials.

**Abstract:**

*Aim*: This literature review presents the biological evaluation of light-curing 3D printing materials containing methacrylic and acrylic resin in dentistry. The sample was 42 articles published between 2008 and 2025, available on PubMed, Scopus, Cochrane, and Google Scholar. The articles were analyzed following the assessment requirements of ISO 10993-2018 (Endpoint) regarding the biological evaluation of each Medical Device. The first selection criterion of the articles was based on the PRISMA schema, concerned with the application of these materials in various fields of dentistry used in 3D printing (e.g., material for crowns and bridges, night, and surgical guide, orthodontic, and denture base). The second criterion included the composition of materials (e.g., catalysts, methacrylic resins, and stabilizers) and the post-curing process. *Results*: The topics discussed in the literature included: (a) estrogenic interactions, sensitization, and the zebra fish model to determine acute toxicity; (b) the main post-processes affecting biocompatibility, i.e., alcohol washing and polymerization in light ovens; and (c) the modification of 3D resins using various types of nanomaterials. *Conclusions*: 3D resins can be used safely in dentistry to make various types of restorations, provided that the polymerization, washing with alcohol and post-polymerization in a light oven follow the manufacturer’s specifications.

## 1. Introduction

The use of 3D printing is becoming popular in dentistry. It may soon overtake currently used technologies such as traditional thermally polymerized acrylic resins or CAD/CAM milling technology from PMMA disks [1,2]. The works performed using this technology include bite splints, surgical guides, and individual impression trays. Additive manufacturing has many advantages, such as smaller material losses in relation to CAD/CAM, the possibility of designing several restorations at once and then printing them at the same time.

This technology can be categorized into two main groups: material extrusion and vast photopolymerization. Material extrusion, like Fused Deposition Modeling (FDM), extrudes heated material to build objects layer by layer. Vat photopolymerization, including stereolithography (SLA) and Digital Light Processing (DLP), uses lasers or projectors to cure photopolymers in a vat to create objects [1,2,3].

Since this technology differs from the traditional ones, it is necessary to use adequate resins intended for printing. It should be noted that many 3D printing materials contain traditional resins verified in composite materials intended for use in a dental office. These include, among others, 2,2-bis(4-(2-Methacryl-oxyethoxy) phenyl) propane (bis-EMA), urethane dimethacrylate (UDMA), triethyl glycol dimethacrylate (TEGDMA), and 2-hydroxyethyl methacrylate (HEMA).

However, new chemical substances such as modern organophosphate-based photoinitiators or compounds with the acrylate group are now used as 3D printing materials [4,5]. There are several types of urethane dimethacrylate, which are oligomers other than di-HEMA trimethylhexyl dicarbamate, which is commonly used in composite materials for fillings in teeth.

In the literature, it is stated that acrylates can be more toxic than methacrylates [6]. Despite the obligation of manufacturers to present chemicals in safety data sheets, including which compounds were used in the delivered material, not all manufacturers post such information. Some safety data sheets may contain residual information on products such as acrylates and acrylic oligomers, without CAS numbers, which is information that allows the user to determine what substance is in the material. What is particularly important in cases of allergic reactions or other adverse reactions to the materials? Therefore, it seems crucial to conduct independent studies, which allow assessing the safety and effects of the 3D printing resins on the human body.

This topic has been recently discussed by many studies [4,5,7]; however, comprehensive review papers are scarce. The literature on the biological evaluation of 3D materials was reviewed only with the focus on cytotoxicity assessment. The reviews [7,8] attempted to present another requirement of biocompatibility for medical materials, that is, the possibility of irritant and sensitizing effects or allergic reactions, and a division of 3D printing materials into the influence of individual chemical components (i.e., resin parts, stabilizers, and polymerization catalysts), which can affect their cytotoxicity or its lack. Moreover, the work by Yüceer et al. presents the latest achievements about 3D printing polymerization methods [9].

Previously, a large part of the materials for 3D printing was classified as Class 1 medical products (i.e., short-term contact with the human body for less than 30 days). At present, more materials with long-term contact (Class 2A) are available on the market. They are intended to produce permanent crowns, removable dentures, or teeth for prosthetics [8]. Materials with long-term contact—externally communicating medical devices—according to ISO 10993-2018, require broader evaluation, which includes cytotoxicity tests, sensitization, implantation and genotoxicity, sub-chronic and chronic toxicity, and carcinogenicity [10,11,12,13,14,15].

Considering the importance of material safety, this article reviews works on the assessment and biological safety of methacrylic resins intended for 3D printing in dentistry, published in the years 2008–2025.

A manuscript that presents the results of a series of studies on various materials, such as their chemical composition and impact on specific aspects of biocompatibility, is more suitable for a narrative review, especially since authors describing cytotoxicity often raise the possibility of genotoxicity in their reports. Some issues, such as allergic reactions to 3D printing materials, are very sparsely described in the literature. Therefore, this topic cannot be compared across multiple publications. However, based on analogues of other materials with similar chemical structures, the occurrence of the described reactions can be expected.

We hypothesize that materials used in 3D printing technology contain methacrylic resins and have a different impact on the human body, which depends on their composition and polymerization conditions.

## 2. Materials and Methods

The analysis concerned the articles from 2008 to 2025 available on the PubMed, Scopus, Cochrane, and Google Scholar databases. The topics for the review included: the materials for the production of individual types of restorations (crowns and bridges, surgical patterns, bite splints, and removable dentures) and related biocompatibility; evaluating cytotoxicity, irritation, and potential allergic reactions; and effects of polymerization and post-polymerization on the properties of individual materials, depending on the degree of conversion (DC) of methacrylic resins.

The following keywords were applied: 3D Printing AND acrylic resin AND Dental AND: cytotoxicity, Genotoxicity, Allergic reaction, Sensitization, Implantation effect, Acute toxicity, Subacute toxicity, Sub-chronic toxicity, Carcinogenetic.

The PECO (Population, Exposure, Comparator, and Outcomes) strategies used for this literature review are presented in Table 1.

The following selection criteria were followed: 3D printing based on acrylic resins used in dentistry, studies on cytotoxicity, post-polymerization method, and adverse reactions after the use of work made using 3D printing technology (Table 2).

Rejection criteria included: the 3D technology other than stereolithography, the resins other than those based on methacrylates or acrylates, incomplete texts, works published in a language other than English, published outside of the identified time range, duplicate/repeated article, medical condition/medical procedure out of scope, not a peer-reviewed article, and similar information is available from a more recent article.

The PRISMA scheme was used for the study.

The authors of the article divided the databases among themselves. MPW reviewed the Scopus and PubMed databases. The second author, ZR, examined the Cochrane and Google Scholar databases. The databases mentioned were checked in the first two weeks of November 2025.

In the first stage, after reviewing the databases, removal of duplicates, and rejection of non-relevant publications based on review of titles and abstracts, articles that did not contain keywords were removed. In the next stage, publications in English were selected. After preparing a list of initially accepted articles, the authors together selected publications that met all the criteria outlined above.

In the event of disagreement regarding a specific publication, the decision to accept or reject it was made through joint discussion between the authors.

### Statistical Analysis

**Figure 1:**
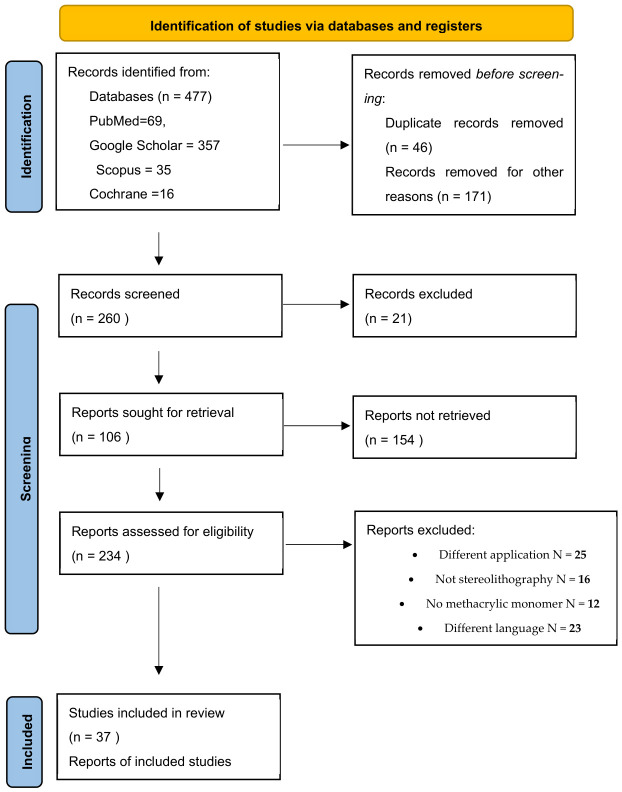
The flow diagram shows the literature review, search strategy, and selection process for including studies (the PRISMA 2020 flow diagram templates are distributed in accordance with the terms of the Creative Commons Attribution (CC BY 4.0) license).

**Figure 2:**
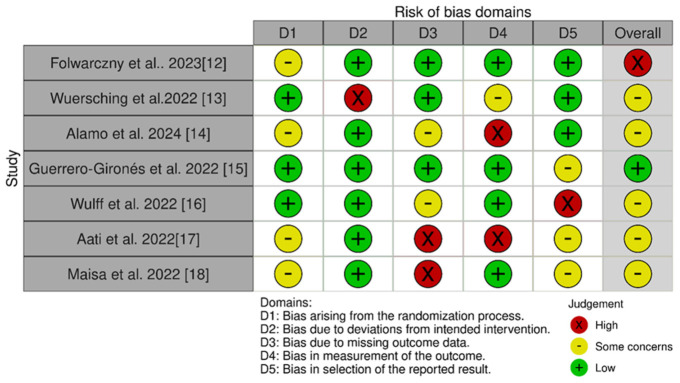
Results of quality/risk-of-bias assessments for 3D printing materials used in various clinical applications. Generated based on the publicly available ROB 2.0 Robvis program [12,13,14,15,16,17,18].

Quality/risk-of-bias assessments of the comparative effect of different groups of materials such as crowns/bridges, night guards, and denture bases were analyzed using software available online. ROB 2.0 Robvis, https://mcguinlu.shinyapps.io/robvis/ (accessed on 12 November 2025). The results are presented in Figure 1 and Figure 2 (Section 2).

## 3. Results

The results obtained from the database review are presented in Figure 1.

The 3D printing resins have been discussed in many articles, with the focus on temporary crowns and bridges, permanent crowns, surgical guides, soft and hard aligners, removable orthodontic apparatus, denture teeth, and removable dentures. Product safety information concerning individual material groups includes materials for making crowns and bridges, night guards and occlusal splints, materials for removable denture bases, catalyst-related cytotoxicity, cytotoxicity of methacrylic and acrylic monomers, inhibitors, post-processing, properties of modified 3d resins, estrogenicity, allergic reaction, sensitization, and acute toxicity.

### 3.1. Materials for Making Crowns and Bridges

Crown and bridge (C&B) materials can be divided according to time of contact with the patient: temporary (below 30 days) or short permanent materials up to one year: Temporary C&B (FL, Formlabs, Somerville, MA, USA) and C&B MFH (ND, Nextdent, Soesterberg, Holland). However, materials with a longer clinical period of use are now available, such as Crowntech Saremco (Rebstain, Switzerland). These materials have a higher content of fillers in the form of silica or glass, which makes their properties like traditional composite filling materials. The colors of the individual materials correspond to the Vita shade guide of the most popular colors: A2, A3, B1, Bleach, and C1. According to the manufacturers, these materials have been tested for biological safety and are not cytotoxic, toxic, or irritating [11]. Materials for making temporary crowns and bridges, such as GC Temp (GC, Tokyo, Japan), contain UDMA, TEGDMA, bis-EMA. In contrast, the SDS card for 3Delta temp (Deltamed, Freiberg, Germany) does not provide information on the monomer content.

Freerprint Temp (Dentax, Ettlingen, Germany) material contains bis-EMA, UDMA, 1,6-hexanediol dimethacrylate, HEMA and TPO. Studies by Folwaczny et al. tested human periodontal ligament (PDL-hTERT) cells exposed to extracts from these materials for 1, 2, 3, 6 and 9 days [12]. To determine the viability of the cell cultures, cell cytotoxicity tests were performed based on the reduction in tetrazolium salts by mitochondrial dehydrogenases of living cells to soluble, orange formazan. Additionally, the authors determined the expression of interleukins IL-6 and IL-8 in the tested supernatants, which are measured in cases of inflammation, and the immune response using ELISA, compared with the control group. Immunofluorescence staining was performed to determine IL-6 and IL-8, as well as scanning electron microscopy of the 3D resin disks tested after contact with the cell cultures. The results of the obtained tests indicate a reduction in cell viability in the case of 3Delta Temp. On day 1 and day 6 of the tests, the 3Delta Temp material caused a significant reduction in both pro-inflammatory mediators. The authors of this article clearly point to the monomers present in this material as an explanation for this phenomenon. 2-hydroxyethyl methacrylate (HEMA) and Tri ethylene glycol dimethacrylate (TEGDMA) might impair cell functions in terms of DNA damage and an altered ability to avert bacterial challenges.

Wuersching et al. studied the 72 h continuous cell viability assay, measuring the reducing potential of the human gingival fibroblast cells with the eluate of the methacrylic materials [13]. Additionally, they determined the cellular inflammatory response in terms of IL-6 and PGE2 levels using specific ELISAs. Oxidative stress was determined by measuring oxidized glutathione concentrations after exposure to the resin eluates. A luminescence-based apoptosis assay was used to detect apoptosis for crown and bridge materials (VarseoSmile Crown plus (NextDent C&B MFH), VarseoSmile Temp (Bego), Temp PRINT (GC), P Pro Crown & Bridge (Straumann). Composite materials in the form of blocks for the CAD milling technique, CAMTetric CAD (Ivoclar) and Telio CAD (Ivoclar), were tested as a comparison group for 3D resins. The results obtained in these tests indicate that VarseoSmile Crown plus and P Pro Crown & Bridge significantly enhanced PGE2 levels. VarseoSmile Temp and P Pro Crown & Bridge demonstrated higher concentrations of oxidized glutathione formed after neutralizing free radicals. All the tested 3D printing resins were slightly responsible for cell apoptosis. Prolonged elevated IL-6 levels, which are induced directly by certain materials, may be responsible for the risk of developing inflammatory diseases such as gingivitis and periodontitis. It should be noted, however, that none of the tested resins significantly increased IL-6 levels in hGF-1. However, the data showed that VSC and P stimulated the production of prostaglandin 2, an eicosanoid mediator produced from arachidonic acid by cyclooxygenases (COX-1 and COX-2) and PGE synthases. The explanation for this fact can be found in earlier studies where bisphenol A-glycidyl methacrylate (Bis-GMA), one of the main monomers used in composite materials, leads to an intracellular GSH depletion in human fibroblasts. Comparable results were also obtained for 2-hydroxyethyl methacrylate (HEMA), which is a diluent monomer. The authors concluded that materials intended for CAD/CAM technology were characterized by greater biocompatibility in comparison to 3D printing resins, because they are hardened at elevated temperatures and under pressure by the manufacturers. Additionally, they do not contain photoinitiators, which may affect cell cultures.

In other studies, other pairs of 3D-printed resins were tested: PZ-3D (Prizma 3D Smart Print Bio A1 resin; Makertech Labs) and CS-3D (Resin Cosmos DLP Temp A1; Yller Biomateriais). These materials were used to evaluate organotypic keratinocyte models in coculture with gingival fibroblasts (HGFs) in a three-dimensional collagen matrix. Cell culture viability as live/dead ratio, and metabolism were then assessed using Alamar blue staining. The results indicated that resins polymerized for 10 and 20 min induced a mild to moderate cytotoxic effect within one day, similar to traditional PMMA-based acrylic resins. However, full recovery of cell viability occurred only after 7 days of incubation [14].

### 3.2. Night Guards and Occlusal Splints

The 3D printing technology can be used to create splints or guards that protect against excessive tooth abrasion (bruxism). Examples of such materials, widely available on the market, include KeySplint Soft (Keystone Industries), NextDent Ortho Rigid (3D System), and Freeprint Splint (Detax). The KeySplint Soft one, according to SDS, contains 2-phenoxyethyl methacrylate, isobornyl methacrylate, HEMA, and TPO. The Next Dent Ortho Rigid contains bis-EMA and Phenylbis(2,4,6-trimethylbenzoyl) phosphine oxide. The Freeprint Splint (Dentax) contains urethane acrylates, Tri propylene glycol diacrylate, and tetrahydrofurfuryl methacrylate and TPO as a catalyst [15].

Guerrero-Gironés et al. described a series of studies conducted by direct contact and 1:1, 1:2, and 1:4 extracts from the above-mentioned materials (Key Splint Soft (Keystone Industries), NextDent Ortho Rigid (3D System), and Freeprint Splint (Detax). Human gingival fibroblasts (hGFs) were selected as the cell model. These were isolated from the gingival tissues. The samples were then examined using MTT assays to assess cellular metabolic activity. Cell migration assays, cytoskeletal staining (identification of fibrous protein structures), cell apoptosis, and intracellular reactive oxygen species production were also used. Additionally, the samples were examined using scanning electron microscopy. After polymerization, the material samples were washed twice in isopropanol and left for 30 min to remove any residual alcohol. Subsequently the samples were cured using a light wavelength of 405 nm, with power intensity of 40 mW/cm^2^ and temperature control condition (25 °C). The results of the MTT test showed that Freeprint Splint significantly reduced the hGF metabolic activity, and the image obtained by SEM analysis showed almost no cells adhered to its surface. Cell migration, tested as wound healing, was significantly lower after exposure to undiluted extracts of Freeprint Splint for 48 and 72 h. Phalloidin staining was performed to observe any changes in cell morphology and in the content and organization of the F-actin (filamentous form of actin) cytoskeleton. The results showed lower adherent cell numbers for Freeprint Splint in extracts at 1:1 and 1:2 dilutions. HGF (hepatocyte growth factor) 2 viability and detection of intracellular reactive oxygen species (ROS) production after exposure to different 3D resins used in dental splints were analyzed using anectin V/7-aminoactinomycin D staining and the general oxidative stress marker CM-H2DCFDA staining, respectively. The authors report that only cells exposed to Keysplint Soft extracts showed similar viability to cells in the control group. ROS levels in undiluted extracts from all resins were higher than in the control group [15].

Wulff et al. investigated in their work other pairs of 3D-printed resins: Luxaprint OrthoPlus (DMG) and V-Print Splint (Voco) [16]. Printing was performed under 90°, 45° or 0° alignment to the building platform. The results indicated that the cytotoxicity of the materials was 9.1 ± 1.3% (printing at an angle of 45°, material V-Print Splint, Voco, manual washing of the sample) and 58.5 ± 5.9% (polymerization at an angle of 90°, material Luxaprint OrthoPlus, automatically washed). At the same time, no difference in cytotoxicity was found depending on the angle of the sample position during printing. The differences in the degree of impact on cell cultures can be attributed to both the thoroughness of the elution of unpolymerized monomers with isopropyl alcohol solution and the difference in the composition of the raw materials (V print splint contains approximately 10% TEGDMA and 10% HEMA, low molecular weight monomers), which have a greater cytotoxic potential in relation to cell cultures.

### 3.3. Materials for Removable Denture Bases

Materials for removable denture bases in the reviewed literature mentioned Dentca Denture Base II (Dentca, Torrance, CA, USA) [17]. It was cured in a Kulzer 3D printer (Cara Print 4.0 Pro) (Kulzer, Hanau, Germany) and was washed with an ultrasonic batch with isopropanol. The samples were then placed in a glycerin solution and cured for various times of 0, 5, 10 and 20 min using a 200 W light curing device (HiLite Power 3D; Kulzer, Hanau, Germany), with a curing light wavelength range of 390–540 nm. Human periodontal ligament fibroblast (ScienCell USA, Carlsbad, CA, USA) samples were kept in direct contact for 48 h. In addition, 50% extracts of post-cured resin were used in the study. As a result, the samples without the post-curing process reduced cell viability by approximately 50% (*p* > 0.001) compared with controls, regardless of whether the resin extract was taken after 24 or 72 h. After resin curing for 5 min or longer, extracts made with this resin were not associated with any cytotoxic effects. All the samples that were in direct contact with cells showed cytotoxic activity, regardless of the post-cure polymerization time [17]. The composition of the material can explain this finding—it contains methacrylate monomer (Proprietary), urethane dimethacrylate and low molecular weight Trimethylolpropane trimethacrylate (concentration < 10%).

Maisa et al. tested NextDent Base (Nexdetn) resin and Dental LT clear resin (Formlabs) and compared them to heat-polymerized acrylic resin samples [18]. After 168 h, there was a non-statistically significant change in cell viability between groups.

The second line of biocompatibility selection is the components used for the 3D printing technology. Here it is possible to distinguish the impact of catalysts and the influence of individual resins as components of the biological safety of the material.

Table 3 below presents 3D printing materials along with their composition and cytotoxicity test results obtained by various authors.

A comparison of the individual studies is presented in Figure 2.

### 3.4. Catalyst-Related Cytotoxicity

Kim GT et al. addressed the issue concerning the catalyst used for polymerization, namely a significant impact on the cytotoxicity of the material. Unreacted photoinitiator may leach out of the polymerized resin when using the material in the oral environment [19].

The purpose of the catalyst used in the 3D resins is to initiate the polymerization reaction by breaking the C=C double bonds in the methacrylate monomers. Their low degree of conversion (DC) during this reaction has a negative effect on the biological, physical, and mechanical properties of the polymer and determines the durability of the reconstruction. The DC depends on several factors, such as the type of methacrylate monomer used (i.e., their molecular weight, viscosity, number of double bonds, temperature of curing process, light intensity, and the wavelength used in the 3D printer). The type and concentration of the photoinitiator system are paramount. Additionally, the light wavelength should correspond to the maximum absorption of the given catalyst [20].

Currently, the most popular catalysts are: TPO-L (ethyl (2,4,6-trimethylbenzoyl) phenylphosphinate), phenylbis (2,4,6-trimethylbenzoyl) phosphine oxide (BAPO) and diphenyl (2,4,6-trimethylbenzoyl) phosphine oxide (TPO). They are considered most accepted for the light-curing process. Their concentration can range from 0.1 to 2 mol% in total resin. The study of Kim GT et al. used the same organic matrix, but the catalysts were changed [19]. The material was hardened in a 3D printer and then post-cured at 350–500 nm, cured for 15 min and washed with isopropanol. The obtained samples of 10 mm in diameter and 5 mm were subjected to 24 h extraction in artificial saliva at 37 °C. Additionally, different concentrations of pure photoinitiators (1 μM and 50 μM) were prepared, which were used for cytotoxicity tests using the MTT method (L-929 mouse fibroblasts). The highest cell viability for printed 3D resin samples was obtained for TPO-L-(89.62 ± 4.93%), while the BAPO group had the lowest cell viability (74.16 ± 3.7%). The cell viability of the TPO group was 84.45 ± 3.62%. When dilute photoinitiator solutions were used, all showed no significant differences at concentrations of 1–10 μM. However, there was a significant difference between concentrations of 25 and 50 μM (*p* < 0.05). According to the literature [20], BAPO shows 50–250-fold higher cytotoxicity than CQ (camphorquinone). Cytotoxicity of human oral keratinocytes by more than 50% can be observed at concentrations greater than 10 μM for this new catalyst. Also, BAPO exhibits a more obvious c response of different types of cells (HEK293, LO2 and HUVEC). Intracellular redox homeostasis is defined as the balance between oxidative and reductive processes in the cell, assessed by detecting intracellular levels of reactive oxygen/nitrogen species. In these studies, the photoinitiator BAPO at concentrations greater than 10 μM induced mRNA expression of redox-regulated proteins after 24 h, similarly to camphorquinone, but at concentrations higher than 2.5 mM. Furthermore, BAPO significantly increased the number of micronuclei, but only in V79 cells. This was observed at a concentration of 10 μM. In comparison, CQ had an effect at a concentration of 12 ± 1.25 mM, and the control in 382 medium at 6 ± 3 mM. However, a significant decrease in the proliferation of these cells was also noted. At a concentration of 10 μM BAPO, the observed reduction was 19.8% ± 7.3% compared to the control group. According to the authors, the TPO-L photoinitiator demonstrated excellent biocompatibility, color stability, and acceptable dimensional accuracy in resin-based materials intended for 3D printing. Other tested catalysts, such as 2-benzyl-2-(dimethylamino)-4′-morpholinobutyrophenone, 4,4′-bis(diethylamino)benzophenone (EMK), diphenyl(2,4,6-trimethylbenzoyl)phosphine oxide (TPO), and 2-isopropylthioxanthone (ITX), showed varying degrees of cytotoxicity to four tissue cell types under the experimental conditions at concentrations ranging from 1 to 50 μM. Among the tested light polymerization initiators, two in particular, ethyl(2,4,6-trimethylbenzoyl)phenylphosphinate (TPOL) and methylbenzoylformate (MBF), showed the lowest cellular toxicity [21].

### 3.5. Cytotoxicity of Methacrylic and Acrylic Monomers

Some 3D printing resins use monomers. They have been used in dentistry for composite resins for a long time. Lin et al. studied the properties of various resins containing the most popular monomers bis-EMA, UDMA and TEGDMA [22]. The authors concluded that the percentage of individual monomers might have an influence on cytotoxic properties in extract tests and MTT cell viability tests (murine fibroblasts L929). Specimen composed with 60% Bis-EMA and 40% UDMA had the highest viability of 118.8 ± 5.7%, while specimen (60% bis-EMA, 30% UDMA and 10% TEGDMA) had the lowest viability of 87.1 ± 7.4%. Specimens containing no TEGDMA had higher viability than the specimens containing 10% TEGDMA. However, all the samples had cell viability greater than 70% and therefore could be considered non-toxic from the point of view of ISO 10993-5 (2009). As an explanation for this phenomenon, the authors supported the thesis that the bifunctional monomers used in the study created a densely cross-linked structure that prevented the release of unpolymerized resins from the center of the material.

Tri ethylene glycol dimethacrylate or 2-hydroxyethyl methacrylate is added to 3D printing resins to reduce their viscosity. According to Lin et al. the material that is cured in the 3D printer should have a viscosity no more than 2000 cP [22]. However, some commercial products for printing crowns and bridges with higher filler content have a viscosity of up to 6000 cP. A material with a lower viscosity will have a higher degree of conversion of methacrylate monomers and, therefore, may have lower cytotoxicity [22]. On the other hand, increasing the concentration of TEGDMA may lead to greater cytotoxicity of the materials, because this monomer has a lower molecular weight, which can be more easily washed out of the polymerized material. Regardless of the resins contained in the materials, their safety of use would be influenced by the way they are processed: appropriate design of the restoration, 3D printing, and all post-polymerization processes.

### 3.6. Inhibitors

All acrylate-based resins used in the dental industry require auxiliary compounds to extend resin shelf life. Inhibitors, which are usually quinone-based compounds such as butylated hydroxytoluene, hydroquinone, hydroquinone monomethyl ether, and benzoquinone, prevent spontaneous polymerization during storage. These types of substances are classified as cytotoxic and genotoxic [23,24].

### 3.7. Post-Processing

In contrast to composite materials used directly in the mouth of the patient (when one layer has 2–10 mm), 3D printing resins are polymerized in thin layers of 25–100 microns. Polymerization in thin layers causes the material to be uniformly hardened throughout its entire volume. Additionally, after printing the crown, bridge or denture base, they are placed in isopropanol, where the unpolymerized monomers are removed from the surface of the material. The time of isopropanol washing of printed samples may play a particularly key role. This was determined in the study by Hwangbo et al. [25]. Contact of the material for 3 min showed cell survival at the level of 30%, while 90 min of washing increased it to 75% (human gum fibroblasts).

In another study, Hwangbo et al. used tri propylene glycol monomethyl ether in comparison to the flammable and high vapor pressure of isopropanol. They obtained comparable results of biocompatibility. However, it should be noted that extended contact with the solvent may cause partial deformation or destruction of the printed material. Thus, many manufacturers do not recommend prolonged contact of polymerized samples with an alcohol solution.

Further post-polymerization of 3D printing objects is conducted in light ovens. Some ovens use an elevated temperature of up to 60 °C for additional double-bond conversion inside the material. The goal is to obtain a material with an extremely low content of residual monomers, which are responsible for high biocompatibility [25].

Tera Harz, a Korean company (Seoul, Republic of Korea), presented another method used in the post-polymerization process of materials intended for shaping memory clear aligners used in orthodontics. After polymerization, the orthodontic device is inserted into a centrifuge for 4 min, where, because of the action of centrifugal force, excess unpolymerized methyl monomers are removed from its surface. Then the aligner is placed in a heat oven and polymerized at a temperature of 80 °C in a nitrogen atmosphere (24 min) [26,27]. The cytotoxicity evaluation of this material (Tera Harz TC-85 DAC) was performed on samples cured for 14, 24 and 50 min. The material after polymerization was stored for 14 days in artificial saliva. Subsequently, the supernatant was collected. Human gingival fibroblasts (HGF-1)-CRL2014 were used to evaluate potential cytotoxicity after 72 h of exposure to the extracts. Reduced cell viability was observed for the samples cured for 50 min. The authors concluded that 3D directly printed aligners showed a cytotoxic effect like the thermoformed conventional aligners. Additionally, the polymerization time recommended by the manufacturer should be strictly followed [26,27].

In a clinical situation, diverse types of post-polymerization devices with a high impact on the properties of the same product Nextednt (C&B MFH) were used. Kim et al. printed specimens of Nextednt underwent process using five different post-polymerization devices: D102H (Sona Global, Jamaica Plain, MA, USA); LC-3DPrint box; (NextDent, Soesterberg, The Netherlands), Form Cure; (Formlabs, Somerville, MA, USA), ME (Medusa, custom-made, Guangzhou, China); and MP (MP100; Hephzibah, Cheonan-si, Republic of Korea) [28]. To compare resin polymerization efficiency, the authors measured the light intensity and temperature of each device during testing. The highest light intensity during the curing process was observed in the MEDUS device. The temperature measured inside the device after polymerization increased the most in the LC 3DPrint device and the least in the D1 device. All the samples had cell survival of more than 60%. However, for the D1103H device, it was 96%, whereas for the MP 100, it was 66%. This may be related to both measured parameters: the light intensity and the length of the polymerization cycle, which was 20–60 min. Interestingly, in the case of the Medusa 6 Chip LEDs (200 LEDs per chip, 395–445 nm) device (Guangdong Medusa Medical Device Co., Ltd., Guangzhou, China), the post-curing time was only 3 s, and the cell culture survival rate reached 90%. This may be explained by the fact that an intense light source can cure the resin surface to obtain high DC, preventing the migration of unpolymerized monomers from the sample, which does not adversely affect the cell cultures.

Another way to reduce the cytotoxicity of 3D resins is to immerse printed resin samples in water at 80–100 °C for one to five minutes after the curing process. This is one of the most effective methods for increasing cytocompatibility by washing out toxic substances such as unpolymerized monomers, photoinitiators, and other additives added during the production process. Warm water, through a local increase in temperature and differences in the concentration gradient, improved the diffusion of residual monomers. However, it should be noted that these samples exposed above the glass transition temperature did not undergo deformation [29].

### 3.8. Properties of Modified 3D Resins

Many reviewed articles described the modification of resins for 3D printing by adding diverse types of materials. Biocompatibility can be obtained by adding appropriate components to the resins (bioactive glasses [30], hydroxyapatite [31], fluorine complexes ((4,4-Bis-4-[2-hydroxy-3-(2-methacryloyloxy)propoxy]-phenyl-pentanol-amine)-N,N-diacetic acid zirconium (IV) fluoride complex [32], titanium dioxide (TiO_2_) and micro-fillers of polyetheretherketone (PEEK) [33]. Another group of additives is nanomaterials, which are used to increase the mechanical resistance of the obtained product: graphene [34], zirconium oxide [17], or silica [8]. Some articles described the addition of zirconium or zinc oxide nanoparticles to increase the bacteriostatic properties of the obtained materials. In such a case, testing the biological properties of the material after modification with new substances is recommended. The first screening test should be cytotoxicity.

Such screening was performed by Aati et al. [35]. They used nano ZrO_2_ covered with silane (concentration 1, 3, and 5%) in a 3D printing resin. The results indicated that the addition of these nanoparticles did not affect the cytotoxicity of the material, and the formation of biofilms from Streptococcus mutans and Candida albicans on the surface of the material was significantly limited.

The effect of resin modification with graphene was presented in the work of Janjić et al. on the mouse fibroblast cell line L929. No toxicity was demonstrated for the graphene content of nanoplatelet flakes from the concentration 0.025–1% (cell survival at 90%). Additionally, the resazurin-based toxicity assay was performed to assess the metabolic activity of Glioma stem cells GSC (taken from five different donors) in response to incubation with 3D-printed specimens (cell survival > 80%). The authors agreed that the addition of diverse types of substances intended to modify 3D printing resins did not affect the cytotoxicity of the material, provided it was properly cured by washing with an isopropanol solution and post-polymerization time varied between 10 and 20 min [33].

**Figure 3:**
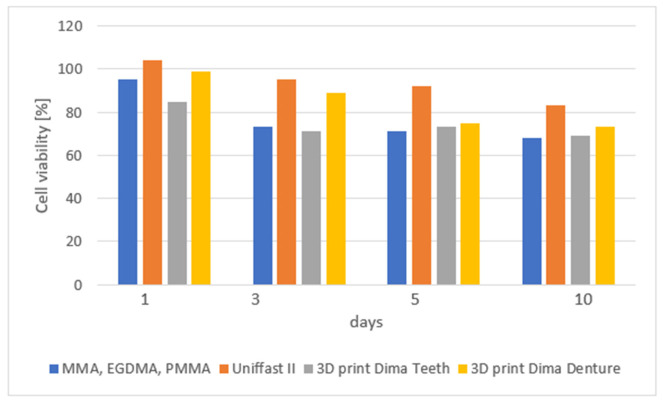
Cell viability in proliferation after 1, 3, 5, and 10 days of the printable PMMA-based resin P30E56M14, conventional PMMA (Unifast II), and commercial 3D-print resins (Dima Print denture base and denture teeth) according to Hata et al. [37].

Hata et al. [36] used mixtures of polymethyl methacrylate, methyl methacrylate, and ethylene glycol dimethacrylate. The authors employed this alternative approach to the composition of materials for 3D printing because they wanted to use raw materials, which are well known and used in dentistry for many years. Forty-two samples were made in the system of these three materials. They were compared to commercial materials for making temporary crowns and bridges, self-curing Unifast II (GC, Tokyo, Japan) and 3D printing Dima Print (Denture Base and Teeth; Kulzer, Hanau, Germany). Cell viability was examined over a period of 1–10 days. These parameters for the PMMA-based resin were significantly lower than those of Unifast on days 1, 3, and 5. These changes could be observed up to 10 days after polymerization. The tested material with a composition of 30% PMMA, 56% EGDMA and 14%MMA was characterized by a similar degree of cytotoxicity > 70% as commercial materials (Figure 3).

### 3.9. Estrogenicity

The bisphenol A-glycidyl methacrylate (bis-GMA resin), commonly used in some materials for dental fillings, is becoming increasingly important in dentistry. This is because the breakdown products of this compound have a structure like the estrogen molecule and can adversely affect the human body. Therefore, many manufacturers are trying to replace this compound with safer alternatives.

The same may also apply to 3D printing resins. Pratsinis et al. [26] tested the Tera Harz TC85A clear aligner resin (Graphy, Seoul, Republic of Korea). Cytotoxicity was determined using the traditional human gingival fibroblast method. Estrogen activity was assessed using the E-screen.26 MCF-7 (estrogen-sensitive) and MDA-MB-231 (estrogen-insensitive) human breast adenocarcinoma cells were grown and subjected for 6 days to samples at a final concentration (*v*/*v*) of 20% in estrogen-depleted medium. 17β-Estradiol (E2; 10^−9^ M) and BPA (10^−8^ M) were used as positive samples. The results indicated that exposure of human gingival fibroblasts to the eluent of Korean 3D-printed aligners did not initiate a cytotoxic effect. Additionally, no oxidative stress was noted, which denotes a lack of long-term effects on the physiology of cells. No xenoestrogenic action was identified, implying the lack of estrogen-like molecules such as bisphenol-A being released from the immersed water aligners. According to information provided by the manufacturer, this product contains a polymer based on urethane methacrylate.

Furthermore, Jung YS et al. [37] tested the following resins: NextDent C&B MFH (3D Systems, Rock Hill, SC, USA), DIOnavi-P. MAX (Dio Co., Busan, Republic of Korea), and DIOnavi-Denture02 (Dio Co., Busan, Republic of Korea). Cytotoxicity was determined using cultured human gingival fibroblasts, and bisphenol A (BPA)-related apoptotic effect using periodontal ligament cells. BPA release was assessed using liquid chromatography/mass spectrometry. Among the materials tested in these studies, no BPA release was observed, suggesting that 3D-printed resin materials do not pose a potential risk of BPA in children.

### 3.10. Allergic Reaction

In the PubMed database, only one article was found on allergic reactions after contact with 3D printing products. The case involved a 60-year-old dental technician who experienced an allergic reaction to methacrylate monomer. As a result, he must abandon his profession, but after a few years, he returned to 3D printing as a hobby, using gloves as appropriate protective measures. Nevertheless, he was diagnosed with erythematous and scaly plaques on his cheeks, eyelids, hands, and forearms. After 14 days of stopping work, these symptoms disappeared. In the 3D printing resin safety data sheet (SDS), it was declared that the product contains acrylic acid, polymeric urethane acrylates, 4-tert-butylcyclohexyl acrylate, and tricyclodecane dimethanol diacrylate. Unfortunately, acrylic compounds used in commercial products are impure and may contain multiple (meth)acrylates, which may not be explicitly stated in the SDS [38].

### 3.11. Sensitization

The 3D printing resins, as well as composite materials, can contain skin irritants and/or sensitizers; however, product safety data sheets (SDSs) might not declare all chemical substances. Bowers et al. [39] characterized elemental and organic skin irritants and sensitizers present in thirty-nine commercial products. The evaluation focused on the influence of resin, manufacturer, system, color, and post-processing on the presence of irritants and sensitizers, and compared product SDSs. The authors identified twenty-three irritant elements, 54 irritant organic substances, 22 sensitizing elements, and 23 sensitizing organic substances (using gas chromatography-mass spectrometry (GC-MS)). The manufacturers provided only information for three to six of these ingredients, respectively. The substances (acetone, isopropyl alcohol, n-hexane, benzene, methyl methacrylate, toluene, ethylbenzene, styrene, m, p-xylene, o-xylene) were selected based on their presence in resins. The results indicated that irritants or sensitizers could be present in the concentration 8.32–4756.65 mg/kg. It was concluded that there was a need for more precise information on these substances in the safety data sheets provided by manufacturers.

Rogers et al. [40] developed the first chip to truck female reproductive tract (EVATAR), to perform sex-based ex vivo research. To increase the scalability and accessibility of EVATAR, the authors tested 3D printing (3DP) technologies, selecting two biocompatible resins (Dental SG (DSG) and Dental LT (DLT)) to generate micro physiologic platforms. Due to the known sensitivity of reproductive cells to leachable compounds, the authors first screened for the toxicity of these biomaterials using an in vitro mammalian oocyte maturation assay. Compounds that can be released from resins (residual monomers, catalysts) can disrupt the maturation process of mammalian oocytes in vitro. The study used mouse oocyte cultures on a cured DSG and Dental LT resins surface intended for 3D printing as a model. Direct contact of cells with the material resulted in their rapid degeneration. However, in samples treated with oxygen plasma, no degeneration was observed, and the majority of the resulting oocytes underwent meiosis in vitro. Detailed microscopic analysis revealed that 57.0 ± 37.2% of cells cultured on DSG resin plates exhibited abnormal chromosome morphology, compared to 19.4 ± 17.3% of control cells cultured in polystyrene. This pattern was observed for all the DLT resins tested, including the plasma-treated samples. Mass spectrometry was used to identify the component that had adverse effects on oocytes. The results indicated that the substance was Tinuvin, a commonly used compound that stabilizes the color of materials exposed to solar radiation. This substance demonstrated a dose-dependent disruption of meiotic progression and an increase in chromosomal abnormalities when exposed to oocytes, which can lead to significant egg toxicity in mammals. The findings from this study clearly demonstrate the potential risks associated with the use of under-tested materials in biomedical applications and underscore the need for more rigorous evaluation of individual components added to materials that are in prolonged contact with living organisms [40].

### 3.12. Acute Toxicity

Mesbah et al. [41], in their study, compared the biocompatibility of methacrylates used in various prosthetic applications, including denture bases, splints, retainers, and surgical guides, using three commercially available materials (E-Denture (ED), E-Guard (EG), and Dental SG (DSG)). A new method based on a 3-day-old zebrafish embryo model was used for the assessment over a four-day period. In direct contact, young fish were exposed to unpurified methacrylates added to ultrapure water at specific concentrations. Fish in the study group showed lower resistance to toxicity compared to fish in the control group. Higher concentrations of toxic extracts added to water induced mainly acute reactions (embryonic mortality), as opposed to cumulative chronic reactions (sublethal and teratogenic effects) [42]. The authors found that the toxicity was mainly due to Ethoxylated bisphenol A dimethacrylate, existing in more than 60% 3D printing resins. This monomer, after polymerization, possesses a lower conversion rate, which can be detected by FTIR spectroscopy [41,42].

The acute and chronic toxicity of six uncured resins to Ceriodaphnia dubia was explored by Ballentine et al. [43]. Two-day acute toxicity (LC50) ranged from 2.6 to 33 mg/L, and inhibition concentration (IC25) values for reproduction ranged from 0.33 to 16 mg/L.

## 4. Discussion

**Figure 4:**
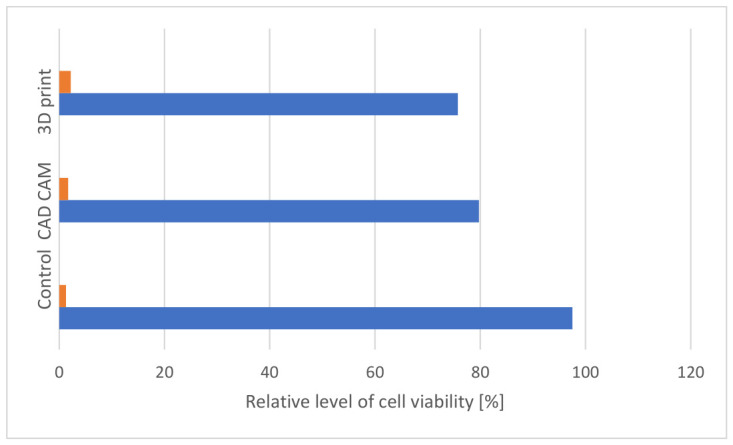
Effect of CAD/CAM and 3D-printed dental materials on OECM-1 tumoral cells viability after 24 h of incubation ± SD compared to control (unexposed cells) according to Enasescu et al. [45] (blue, results, orange color SD).

The hypothesis that was placed at the beginning was confirmed. The review showed that the 3D printing materials from different manufacturers may have quite different biological properties. This depends on their chemical composition, the curing method and post-processing. In the case of cytotoxicity studies, articles discussed studies of 3D printing resins on mouse cells and human gum fibroblasts, OECM-1 tumoral cells. The tests were performed as direct contact or with several types of water extracts, artificial saliva, or organic solvents [44] (Figure 4).

Most authors agreed that the unpolymerized material had strong cytotoxic properties. However, light polymerization and post-curing process allow for obtaining the materials with high biocompatibility, where the cell cultures’ survival rate is over 80%.

In the literature, no articles related to genotoxicity, chronic toxicity, sub-chronic toxicity, subacute toxicity, or carcinogenicity were found. On the websites of some manufacturers, it is possible to obtain information that the products were tested according to the appropriate biological standard and do not have genotoxic or irritating properties. By contrast, some articles determine genotoxicity or irritating effects for free monomers such as TEGDMA [45,46], HEMA [47,48,49] or bis-GMA [50]. Such resins are well known as composite materials for tooth fillings. Therefore, it is important to process the 3D printing material to obtain a product with the lowest content of unpolymerized monomers.

The high cytotoxicity of low-molecular-weight monomers stems from two facts. Photopolymerization causes only partial polymerization of the C=C double bonds in the methacrylate group, reaching 60–90%. When these materials come into contact with the environment (the patient’s oral cavity or cell cultures), these unpolymerized monomers slowly migrate into the surrounding environment. Therefore, many studies demonstrate high cytotoxicity after 48 or 72 h of contact [45].

Catalysts or their decomposition products also belong to low molecular weight substances and, like residual monomers, can be washed out of the material.

In a clinical situation, this may manifest as a superficial allergic reaction, redness, itching, or a painful reaction [19].

All the studies presented in this review were performed as in vitro tests. In vivo materials may interact with enzymatic activity, including the effect of esterases on breaking down composite resins, microbial activity, pH, and temperature fluctuation. Inside the oral cavity, cured resins are also subjected to abrasion and the impact of chemical degradation by consumed food (fats, energy drinks, or alcohol). Therefore, in vitro studies such as aqueous immersion underestimate the effect of environmental factors on the degradation of polymers [51].

For increasing the biocompatibility of 3D printing materials during clinical treatments, the manufacturer’s recommendations are followed (curing time and thickness of a single layer, recommended printer). Post-curing activity, such as washing of the polymerized material with isopropanol using an ultrasonic cleaner, followed by additional polymerization in a light oven are necessary [17,25,27]. Some authors also recommend, similarly to traditional acrylic resins based on PMMA after curing processes, storing materials in distilled water. This allows wash out unpolymerized monomers or catalysts from the material, which can significantly increase the biocompatibility of the printed restoration. It is also important to thoroughly polish the prosthesis to minimize the colonization of microorganisms on its surface [29].

Material manufacturers, therefore, bear a significant responsibility when designing and producing materials for 3D printing. Particular attention should be paid not only to improving the mechanical properties of materials, but also to developing new resins and catalysts characterized by high biocompatibility [8,52].

Therefore, further research on this type of material is necessary. The present review has some limitations—the review of biological properties is based mainly on in vitro studies. Future works should review information related to the studies of 3D printing resins in vivo conditions.

The research was conducted on a specific number of articles, in addition to a variety of test methods, making direct comparisons challenging.

The studies cited above cover a narrow group of 3D printing materials. There is a wide range of resins on the market, but research data for these resins is not widely available. Table 3, which compares the cytotoxicity results of individual materials and their composition, shows that some manufacturers use very different resins. Furthermore, the information contained worldwide is incomplete, and the section on chemical composition often includes proprietary information.

It would be desirable to establish a single research protocol for most 3D printing materials, for example, in the form of an appropriate ISO standard.

The literature analysis conducted will allow us to prepare for future research on the effects of other resins on cell cultures.

## 5. Conclusions

The 3D printing materials are very close in chemical composition to composite resins; improperly polymerized materials have strong cytotoxic properties and can, in rare cases, cause allergic reactions.

To maximize the biocompatibility of 3D printing resins, it is important to thoroughly cure them in strong polymerization lamps and wash them with a propyl alcohol solution to minimize the content of catalyst decomposition products and residual monomers in the entire volume of the material.

## Figures and Tables

**Table 1:** PECO strategy for protocol devised for the review.

Criteria	Description
Population (P)	3D resins used in dentistry
Exposure (E)	In vitro or in vivo assessment: cytotoxicity, cell culture, genotoxicity, allergic reaction, estrogenicity, acute toxicity, subacute toxicity, and sub-chronic toxicity.
Comparator (C)	Comparison of different 3D printing resins with different applications tested under different conditions to evaluate biocompatibility, chemical composition, and post-curing process.
Outcome (O)	Primary outcome was assessment of biocompatibility which was evaluated by cytotoxicity and inflammatory responses.

**Table 2:** Inclusion criteria.

Article Criterion	Options
Study type:	(a) Systematic review or meta-analysis [included] (b) Descriptive review article [included] (c) Clinical trial [included] (d) Well-designed clinical study [included] (e) None of the above [excluded]
Article language:	(a) English [included] (b) Other [excluded]
The article discusses a clinical condition in which the subject device is indicated.	(a) Same condition [included] (b) Minor deviation [included] (c) Major deviation [excluded]
The article provides relevant information regarding technical and/or clinical features of the device group.	(a) Applicable population [included] (b) Limited population [included] (c) Different population [excluded]
The article provides information relevant to a risk/benefit assessment of the subject device technology.	(a) High Quality [included] (b) Minor deficiencies [included] (c) Insufficient information [excluded]
The article contains sufficient information for a rational and objective assessment.	(a) Yes [included] (b) Technique, case series, or case report [included] (c) No [excluded]
The publication date is within the target date range.	(a) Yes [included] (b) No [excluded]

**Table 3:** Comparison of the composition of 3D printing materials used in various clinical applications based on their available chemical composition.

Temporary Crown and Bride Materials			
Autor	Tested Materials	Composition	Cytotoxicity
Folwaczny et al. 2023 [12]	MFH, Nextdent; GC Temp, GC; Freeprint temp, (Detax) 3Delta temp, (Deltamed) Grandio disc, (Voco) Luxatemp, DMG	UDMA, TEGDMA, bis-EMA	severely affect cell viability Grandio induces only minor changes in direct contact with these cells
Wuersching et al. 2022 [13]	VarseoSmile (Bego) Crown plus (NextDent) Temp PRINT (GC), P Pro Crown & Bridge (Straumann) CAMTetric CAD (Ivoclar), Telio CAD (Ivoclar)	Ethoxylate Dimethacrylate Ethoxylate Dimethacrylate (UDMA) TEGDMA Ethoxylate Bis-GMA Cross-linked dimethacrylate resin PMMA	3D-printed resins have higher cytotoxicity compared to CAD CAM materials
Alamo et al. 2024 [14]	PZ-3D (Prizma 3D Smart Print Bio) CS-3D (Resin Cosmos)	Acrylic monomers Tricyclo [5.2.1.0 2,6] decanedimethanol diacrylate, UDMS HEMA	post-polymerization for 10 and 20 min promoted a mild-moderate cytotoxic effect
Night guards and occlusal splint			
Guerrero-Gironés et al. 2022 [15]	Key Splint Soft (Keystone Industries), NextDent Ortho Rigid (3D System), Freeprint Splint (Detax)	2-phenoxyethyl methacrylate Isobornyl methacrylate 2-hydroxyethyl methacrylate Ethoxylated Bisphenol A, Trimethylolpropane UDMA, Tri propylene glycol diacrylate	cell viability similar to control medium cytotoxicity high cytotoxicity
Wulff et al. 2022 [16]	Luxaprint OrthoPlus (DMG) V-Print Splint (Voco)	ethoxylated bisphenol A dimethacrylate. ethoxylated bisphenol A, TEGDMA, HEMA	high cell survival when printing at a 45-degree angle
			high cytotoxicity
Denture base materials			
Aati et al. 2022 [17]	(Dentca Denture Base) Vertex Rapid (Vertex)	UDMA PMMA	no significant difference between heat-cured material and 3D specimens post-cured for 20 min
Maisa et al. 2022 [18]	Dentca Denture Base II (Dentca)	UDMA	samples without the post-curing process reduced cell viability by approximately 50%, 5-time post-processing 90% cell survival

Materials used in various clinical applications such as temporary crowns, night guards, and dentures are composed of certain similar methacrylate monomers, oligomers such as UDMA or bis ethoxylated EMA. Additionally, some contain low-molecular-weight smudging agents such as HEMA or TEGDMA.

## Data Availability

No new data were created or analyzed in this study. Data sharing is not applicable to this article.

## Abbreviations

The following abbreviations are used in this manuscript:

BAPO	phenyl bis (2,4,6-trimethylbenzoyl) phosphine oxide
Bis-GMA	bisphenol A glycidyl methacrylate
Bis-EMA	2,2-bis(4-(2-Methacryl-oxyethoxy)phenyl)propane
C&B	crown and bridge
CAD/CAM	computer-aided design and computer-aided manufacturing
COX-1	cyclooxygenases
DC	degree of conversion
ELISA	enzyme-linked immunosorbent assay
EMK	4,4′-Bis(diethylamino) benzophenone
EVATAR	miniaturized, 3D organ-on-a-chip model of the female reproductive tract
GC-MS	gas chromatography-mass spectrometry
GSC	glioma stem cells
GSH	glutathione depletion
HEMA	2-hydroxyethyl methacrylate
HGF	human gingival fibroblasts
IL	proinflammatory cytokines interleukin
ITX 2	isopropylthioxanthone
MBF	methyl benzoylformate
PEEK	polyether ether ketone
PGE2	prostaglandin E2
PMMA	polymethyl methacrylate
ROS	reactive oxygen species
SEM	scanning electron microscopy
TEGDMA	triethyl glycol dimethacrylate
TPO	triphenylphosphine oxide
TPO-L	ethyl (2,4,6-trimethylbenzoyl) phenylphosphinate)
UDMA	urethane dimethacrylate
XTT	two-reagent colorimetric assay used to assess cell viability as a function of cell number based on metabolic activity
